# Delayed Fracture After Medial Unicompartmental Knee Replacement: Two Cases of Peri-Implant Fracture

**DOI:** 10.7759/cureus.111166

**Published:** 2026-06-19

**Authors:** Zayar Aung, Takafumi Hiranaka, Ryoma Inoue, Yasuhiro Fukai, Motoki Koide

**Affiliations:** 1 Orthopaedics and Traumatology, Nyaung U Hospital, Nyaung U, MMR; 2 Orthopaedic Surgery and Joint Surgery Centre, Takatsuki General Hospital, Takatsuki, JPN

**Keywords:** decreased bone density, delayed fracture, peri-implant fracture, stress shielding, total knee arthroplasty, unicompartmental knee arthroplasty

## Abstract

Unicompartmental knee arthroplasty (UKA) is widely performed for medial compartment knee osteoarthritis due to its benefits of faster recovery, preservation of knee kinematics, and long-term survival. However, complications such as peri-implant fractures can occur. Most fractures are reported within three months postoperatively, but this study highlights two cases of delayed fractures occurring four and seven years after UKA, emphasizing the importance of long-term follow-up.

In Case 1, a 79-year-old woman experienced a tibial peri-implant fracture seven years after UKA. Bone density loss and stress shielding were suspected contributors. Treatment involved conversion to stemmed total knee arthroplasty (TKA), which resulted in fracture union and restored function. In Case 2, a 73-year-old woman developed a fracture four years post-UKA. Following plate fixation and subsequent re-fracture, she underwent stemmed TKA, achieving fracture union and improved knee function.

The exact cause of delayed fractures remains uncertain, but likely factors include age-related bone density reduction and stress shielding due to the implant. Unlike acute fractures, delayed fractures present unique challenges, and internal fixation may not ensure long-term stability. Stemmed TKA provides reliable stability, reduces micromotion, and promotes fracture healing. These cases underscore the importance of monitoring bone quality in UKA patients and considering stemmed TKA as a preferred treatment for delayed peri-implant fractures.

## Introduction

Unicompartmental knee arthroplasty (UKA) is considered to be an effective treatment for single-compartment osteoarthritis (OA) of the knee joint. Preservation of the unaffected contralateral compartment, the patellofemoral joint, and the cruciate ligaments may help to maintain more normal kinematics and function than can be expected with a total knee arthroplasty (TKA) [1-4]. Advantages of UKA include preservation of the intact weight-bearing compartment and anterior and posterior cruciate ligaments [4], which contributes to reducing intraoperative blood loss [5-10], increasing the range of motion and reducing postoperative pain, thereby facilitating early rehabilitation and resulting in low morbidity and mortality [11, 12].

Despite the advantages, some complications specific to UKA have also been reported. In addition to lateral OA progression, other serious complications include bearing dislocation, component loosening, and periprosthetic fractures. The incidence of peri-implant fractures after UKA has been reported as 0%-8%, but unlike lateral OA progression, which tends to occur several years postoperatively, most fractures reportedly occur intraoperatively or within three months of surgery and are nontraumatic [1, 10, 12]. Nonetheless, reports on the complications of UKA, especially tibial plateau fractures, are rare. In this article, we therefore present two cases from our hospital of peri-implant fractures of the tibia that occurred seven years and four years, respectively, after Oxford UKA.

## Case presentation

Case 1

**Figure 1:**
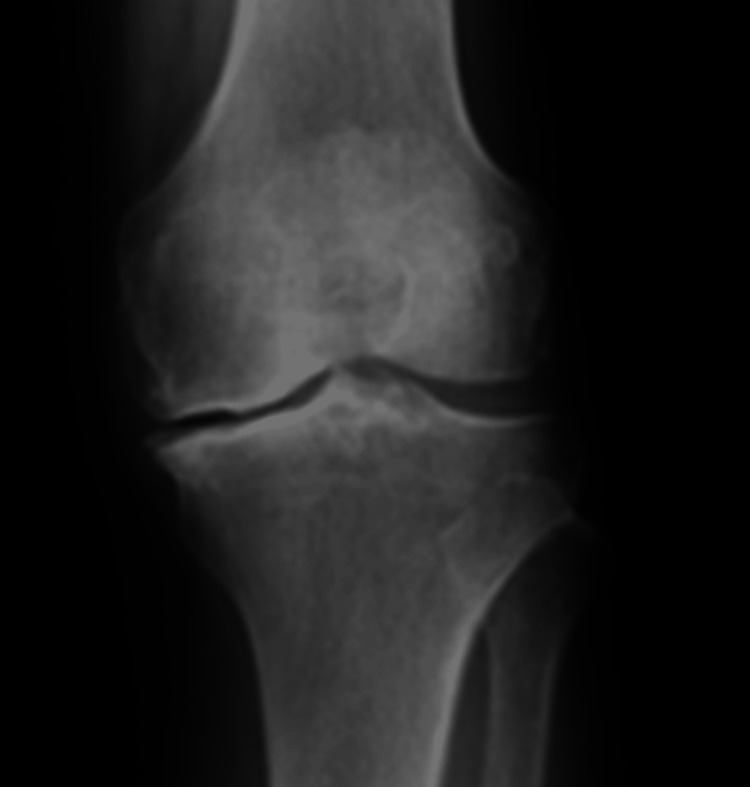
Preoperative radiography

**Figure 2:**
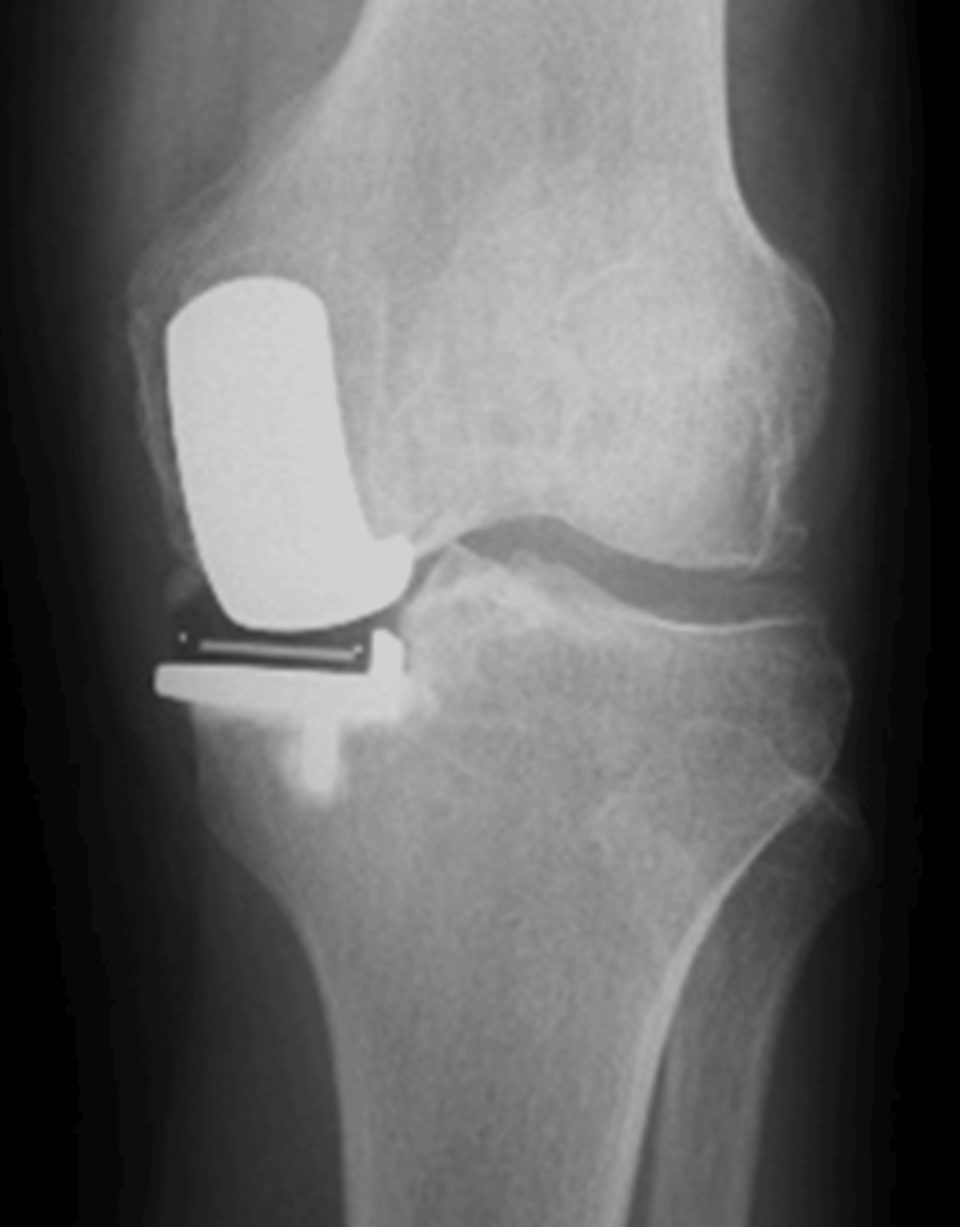
Postoperative radiography

A 79-year-old woman had a 10-year history of chronic left knee pain. Radiologic imaging showed medial joint space loss but an intact lateral compartment as part of degenerative arthritis affecting the medial knee compartment. She had been treated for collagen disease at 35 years of age, and she was diagnosed with type 2 diabetes mellitus at 68 years of age. Cemented UKA (Oxford Partial Knee, Zimmer Biomet, Warsaw, Indiana) was performed using the Microplasty instrument set (Zimmer Biomet) with femoral size S, tibial size A, and mobile bearing size 3 (Figures 1, 2). After surgery, the patient was free of pain and had good function of the left knee.

Six years and seven months after UKA surgery, the patient was diagnosed with lung cancer, for which a right upper lobectomy was performed at another hospital. During that hospital stay, she developed left knee pain without any apparent cause, so she returned to our department approximately seven years after the UKA operation.

**Figure 3:**
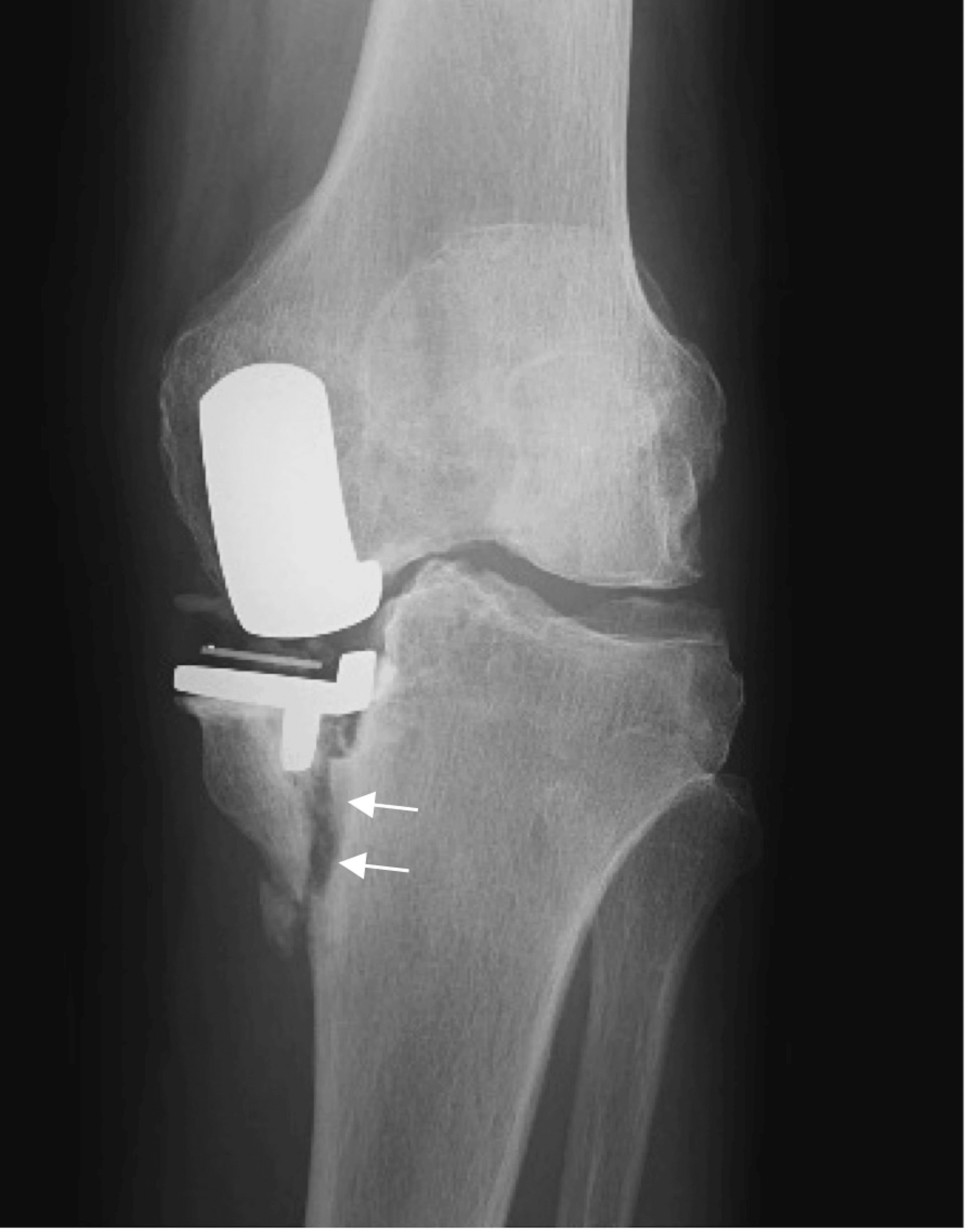
Radiography of the peri-implant fracture

**Figure 4:**
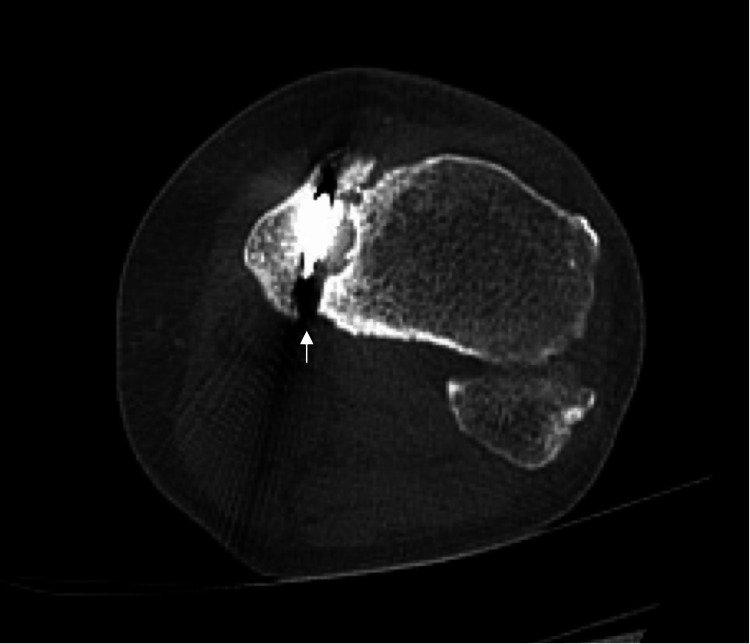
Computed tomography of the peri-implant fracture

**Figure 5:**
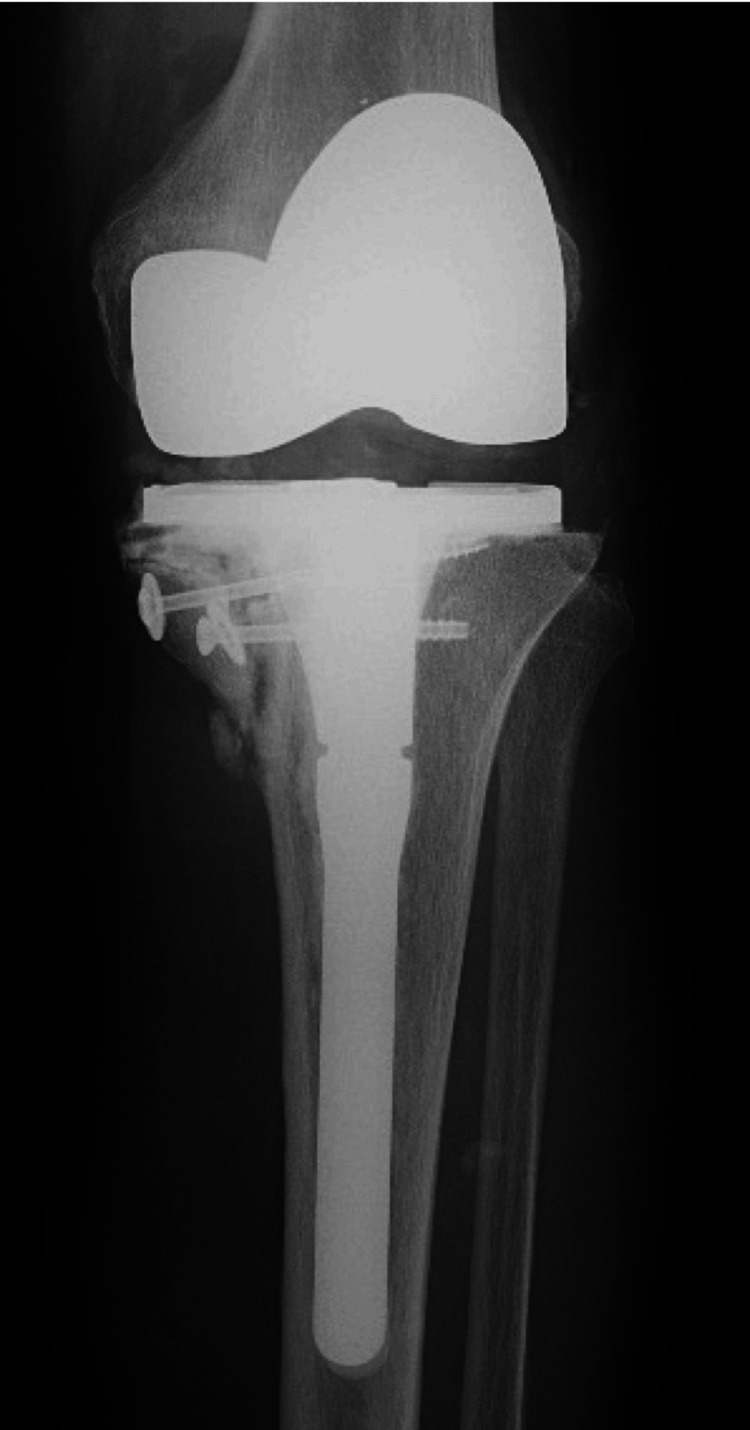
Immediate postoperative radiography of TKA (AP view) TKA: total knee arthroplasty, AP: anteroposterior.

**Figure 6:**
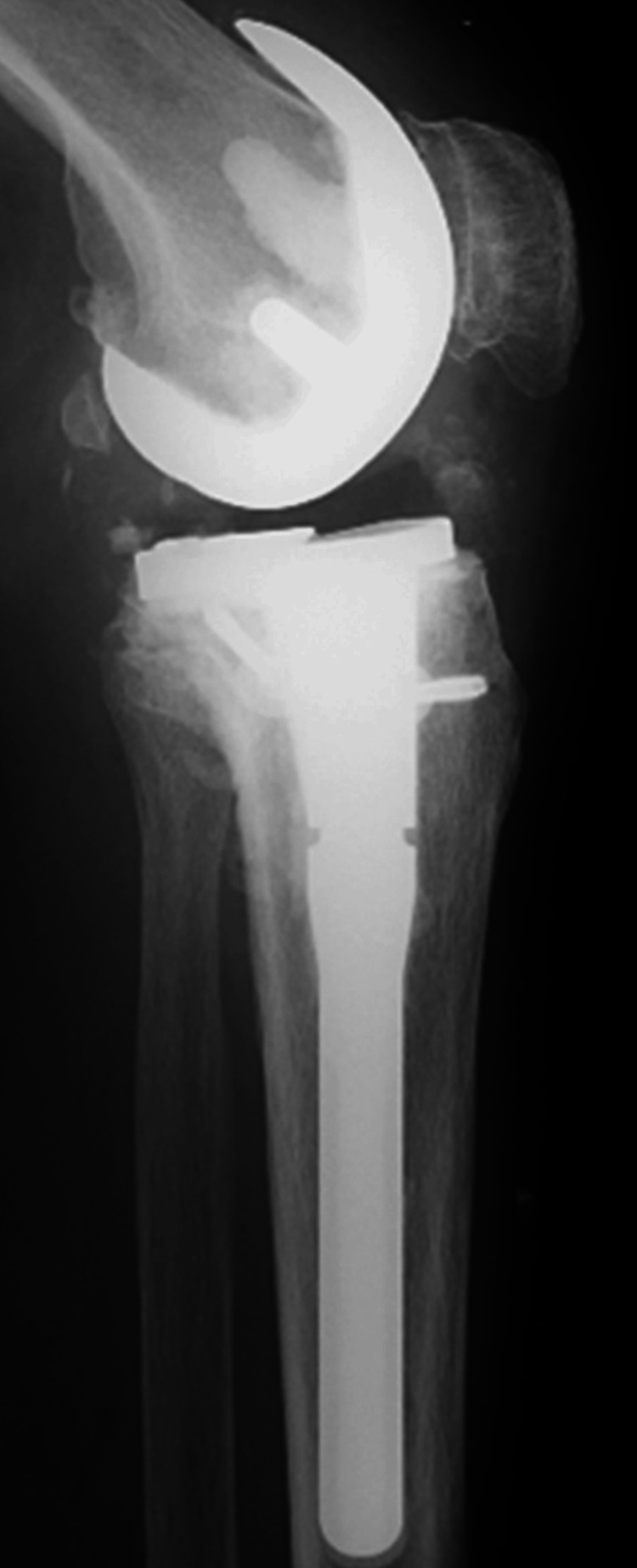
Immediate postoperative radiography of TKA (lateral view) TKA: total knee arthroplasty.

**Figure 7:**
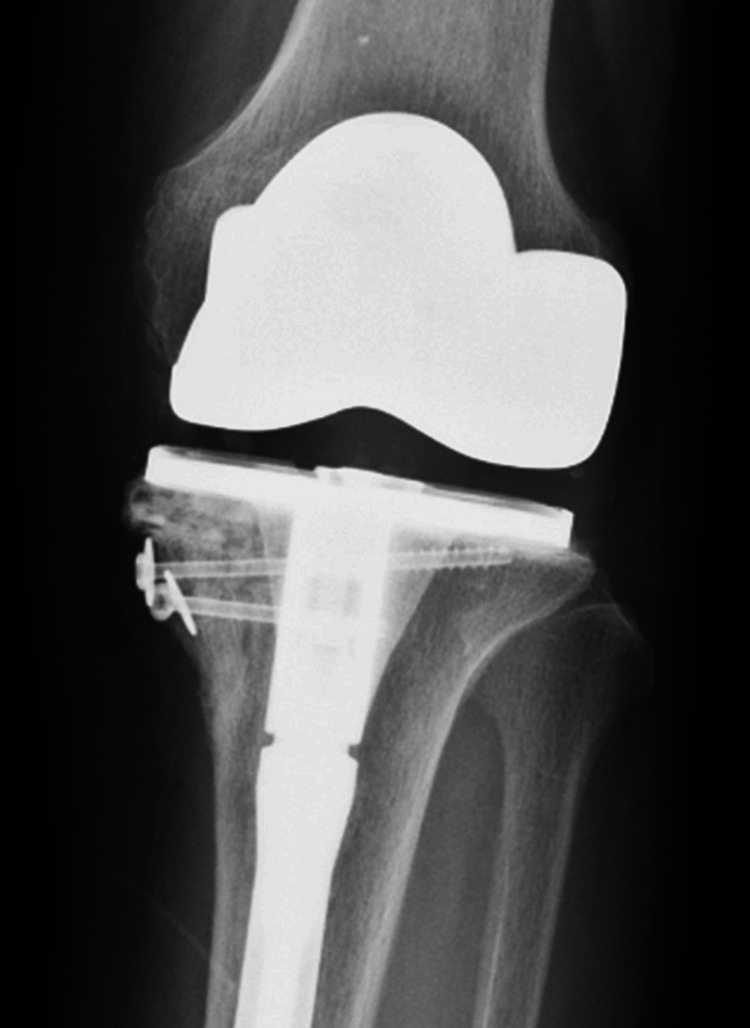
Two years postoperative radiography of TKA (AP view) TKA: total knee arthroplasty, AP: anteroposterior.

**Figure 8:**
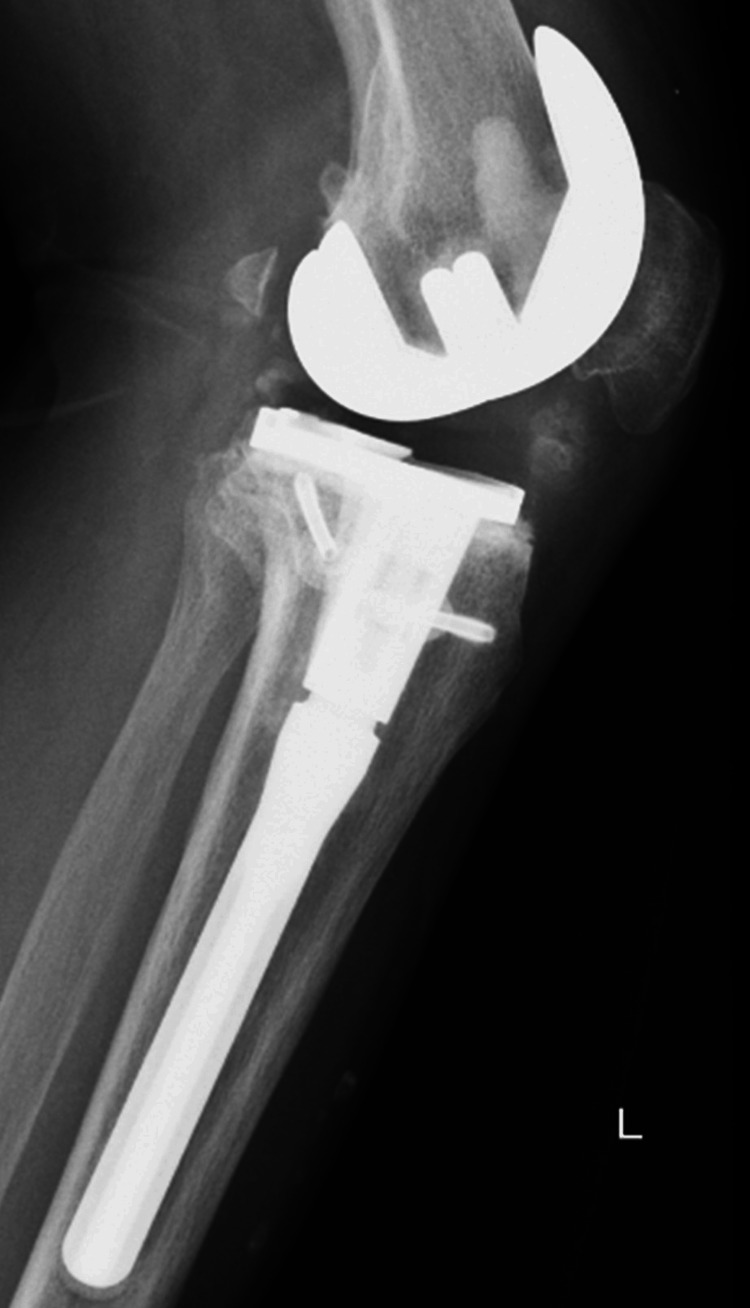
Two years postoperative radiography of TKA (lateral view) TKA: total knee arthroplasty.

Radiography and computed tomography showed a fracture line running downward from the knee area (Figures 3, 4). Radiographs and CT demonstrated a fracture line involving the tibial keel region and extending to the medial tibial cortex, consistent with a medial tibial peri-implant fracture. Because the fracture was already substantially displaced and sclerosis was present at the fracture surfaces, we considered that a considerable period had elapsed since the fracture occurred. Anatomical reduction was therefore expected to be difficult, and reliable bone union with internal fixation alone was considered unlikely. Consequently, conversion to TKA with a stemmed tibial component was selected to bypass the fracture site and provide stable fixation. The patient therefore underwent TKA with a stemmed tibial component (NexGen CR, Zimmer Biomet). No bone loss or loosening was seen on the femoral side. On the tibial side, however, the fracture had not healed and the bone fragment was unstable, so it was fixed with two cannulated cancellous screws (Figures 5, 6). The patient was able to walk with full weight bearing from the day after surgery. Two years after surgery, the patient had 0°-140° range of motion in the left knee, and her Oxford Knee Score was 42. The fracture was shown to be united, and there were no signs of loosening (Figures 7, 8).

Case 2

**Figure 9:**
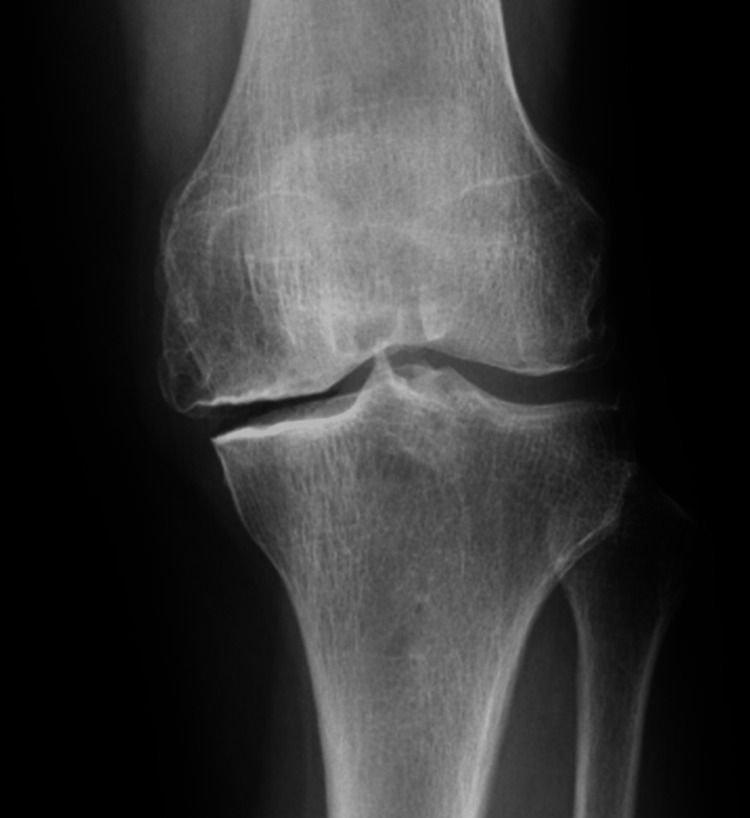
Preoperative radiography

**Figure 10:**
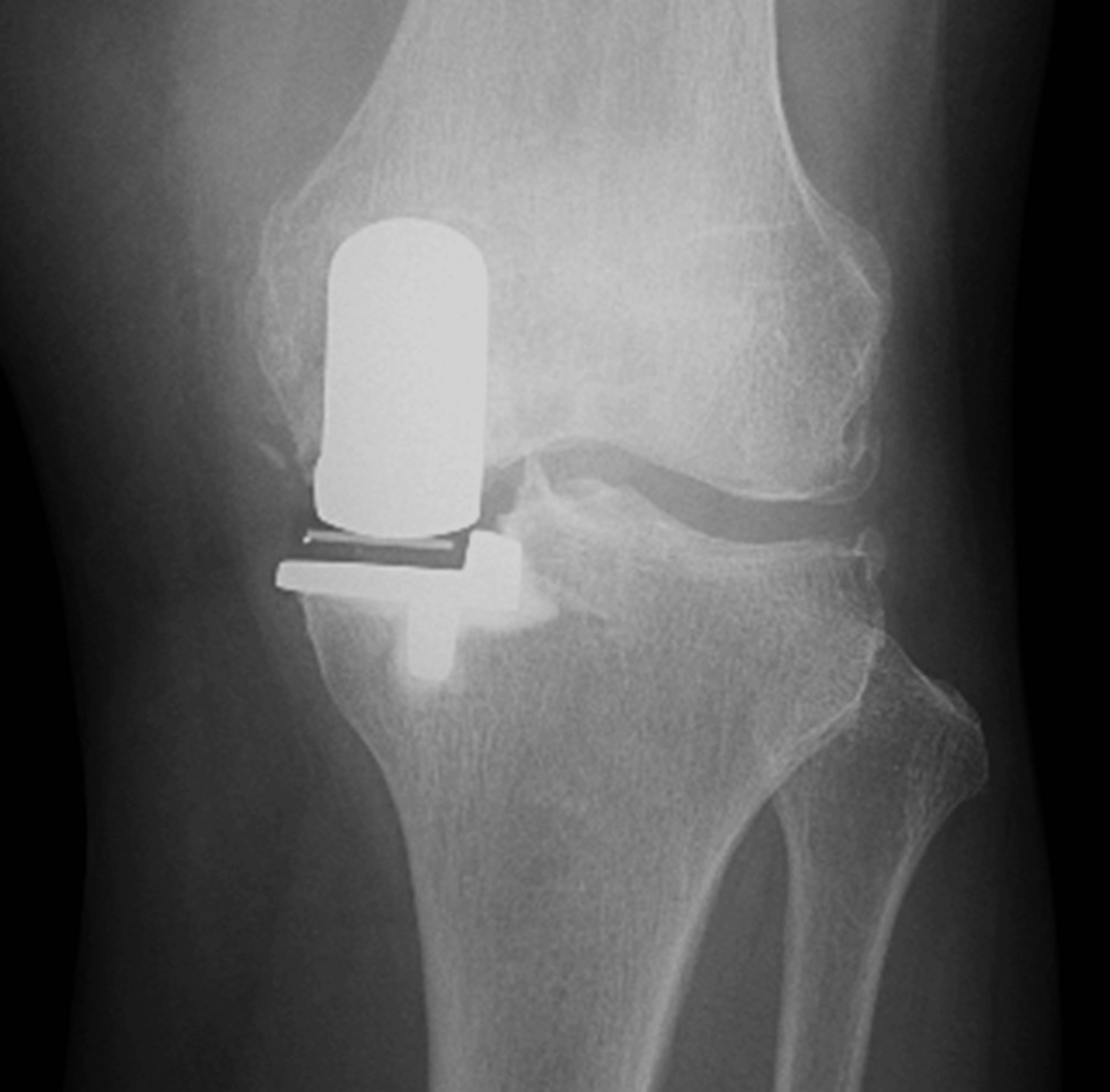
Postoperative radiography

**Figure 11:**
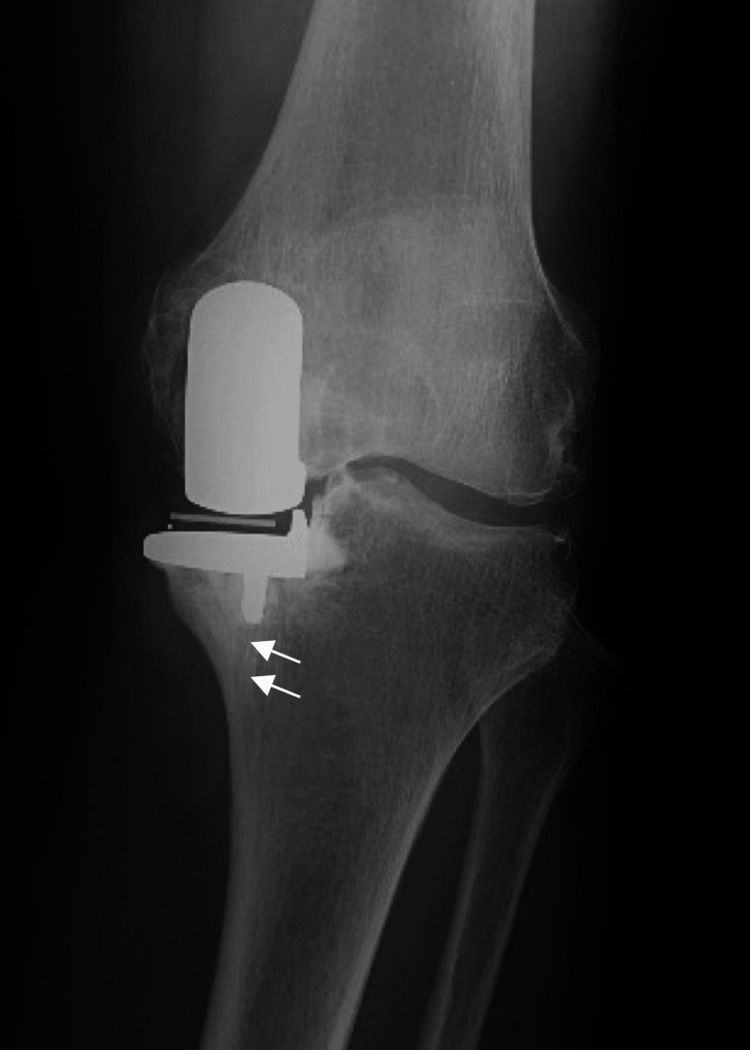
Four years postoperative radiography of the peri-implant fracture

**Figure 12:**
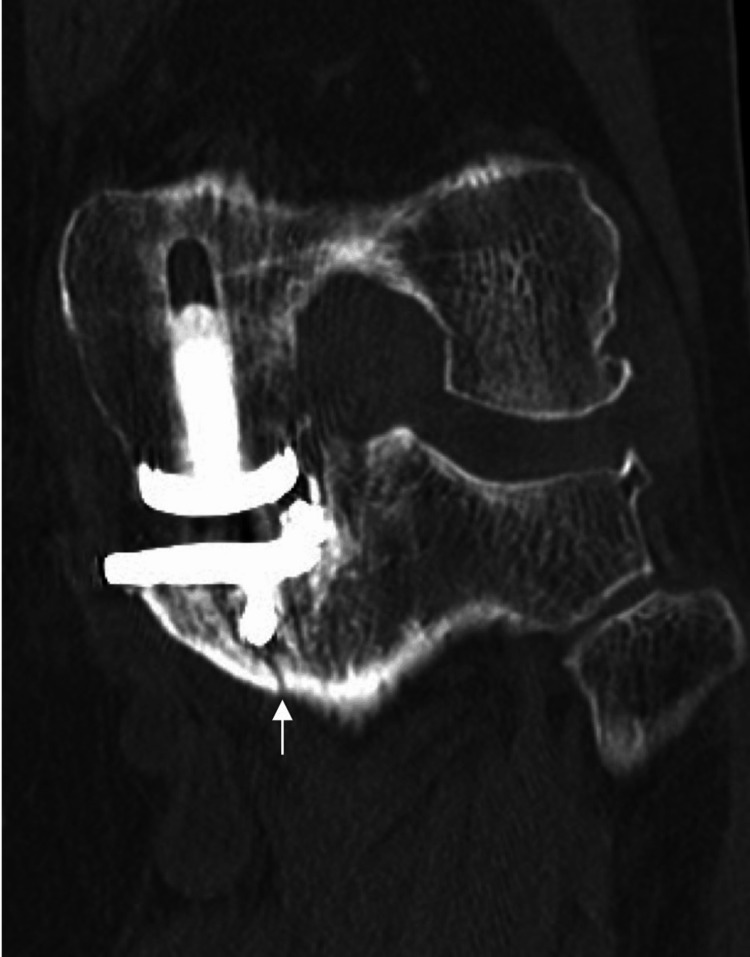
Four years postoperative computed tomography of the peri-implant fracture

**Figure 13:**
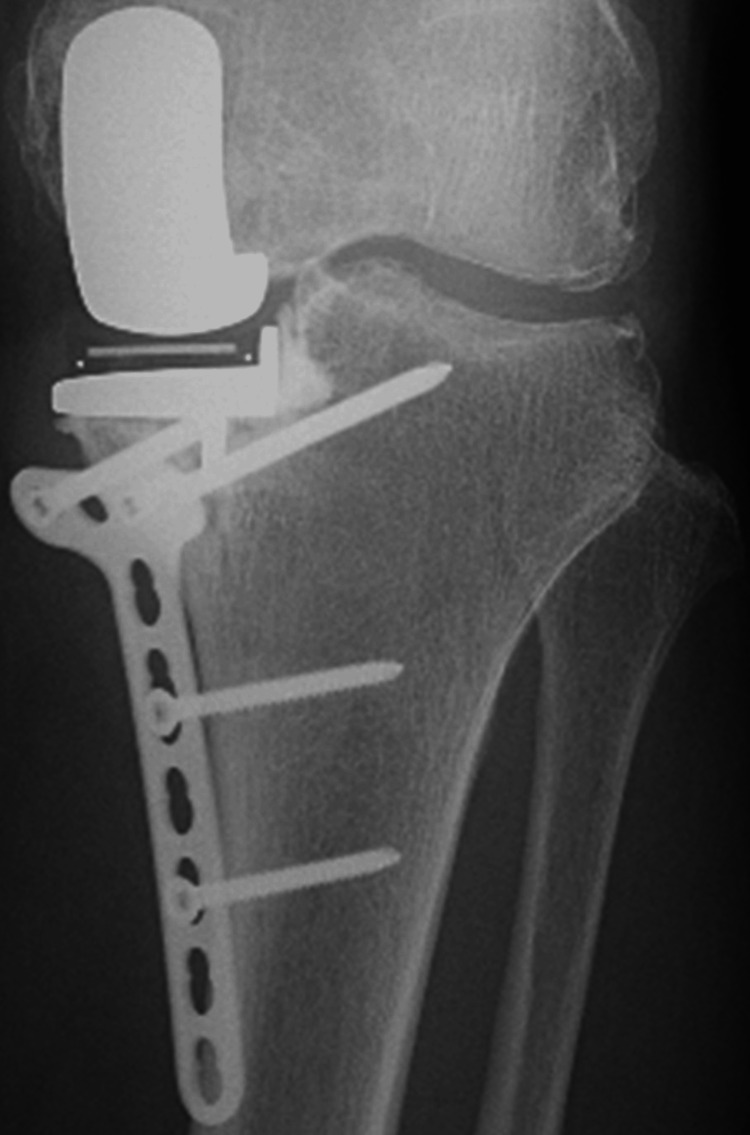
Radiography of reoperation with tibial compression plate (AP view)

**Figure 14:**
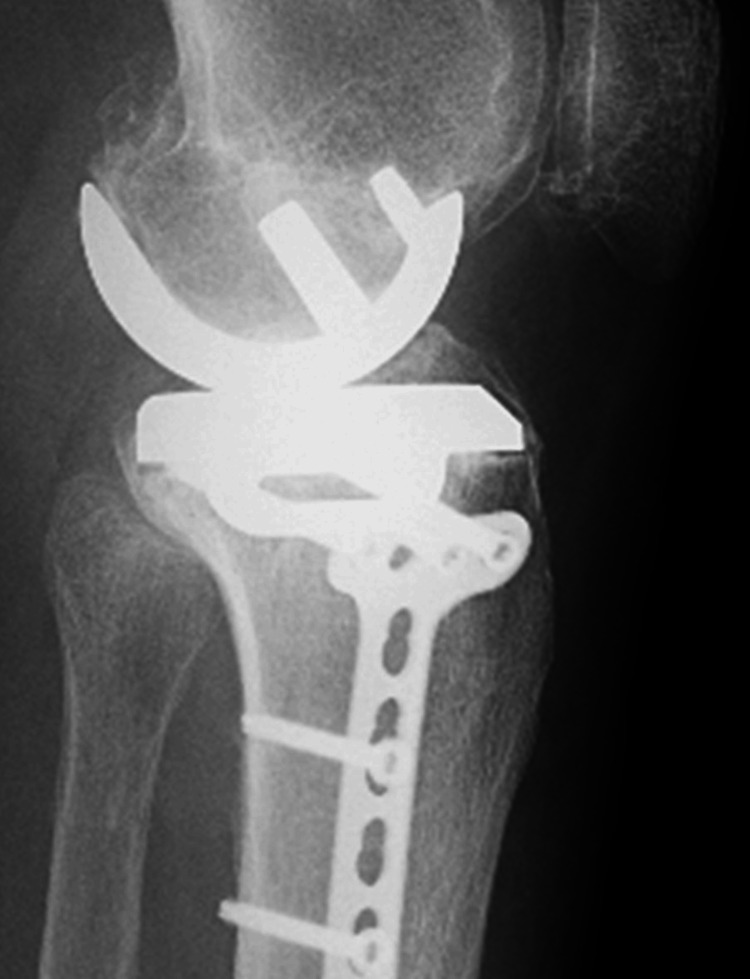
Radiography of reoperation with tibial compression plate (lateral view)

A 73-year-old woman had a 10-year history of chronic pain in both knees. Radiologic imaging showed bone-on-bone contact in the medial joint space. Comorbidities included hypertension and stented coronary artery disease, and she was taking teriparatide for osteoporosis. However, detailed bone mineral density values and the exact duration of osteoporosis treatment were not available from the medical records. Cemented bilateral UKAs (Oxford Partial Knee) were performed using the Microplasty instrument set with femoral size S, tibial size A, and mobile bearing size 3 (Figures 9, 10). The postoperative course was uneventful, but 4 years later, the patient developed left knee pain without any apparent cause and presented to our department. Radiography and computed tomography showed a fracture line running from the keel lesion to the medial metaphyseal cortex (Figures 11, 12). The displacement was minimal, so we performed open reduction and internal fixation using a tibial buttress plate with locking screws (Figures 13, 14).

**Figure 15:**
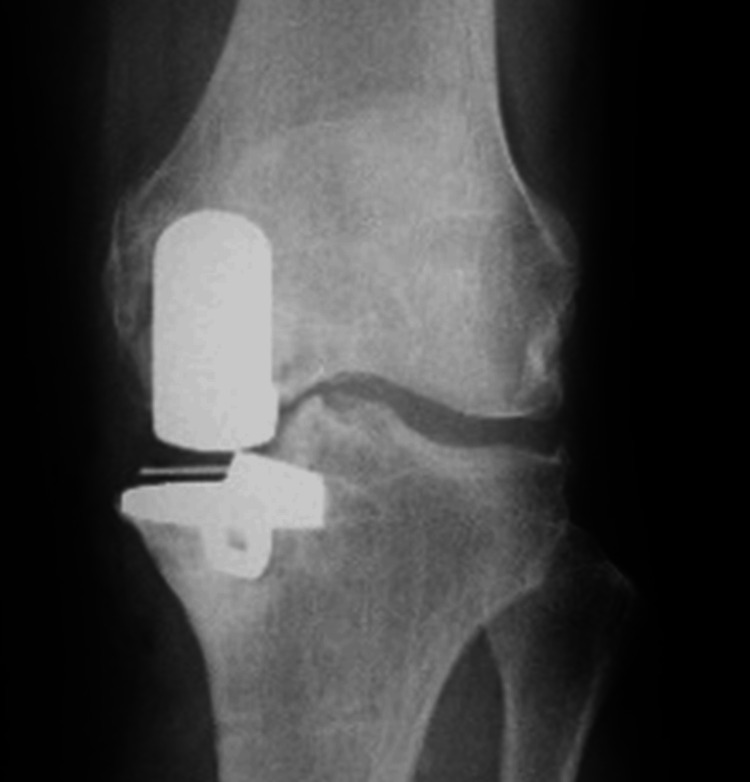
Postoperative radiography after plate removal (AP view)

**Figure 16:**
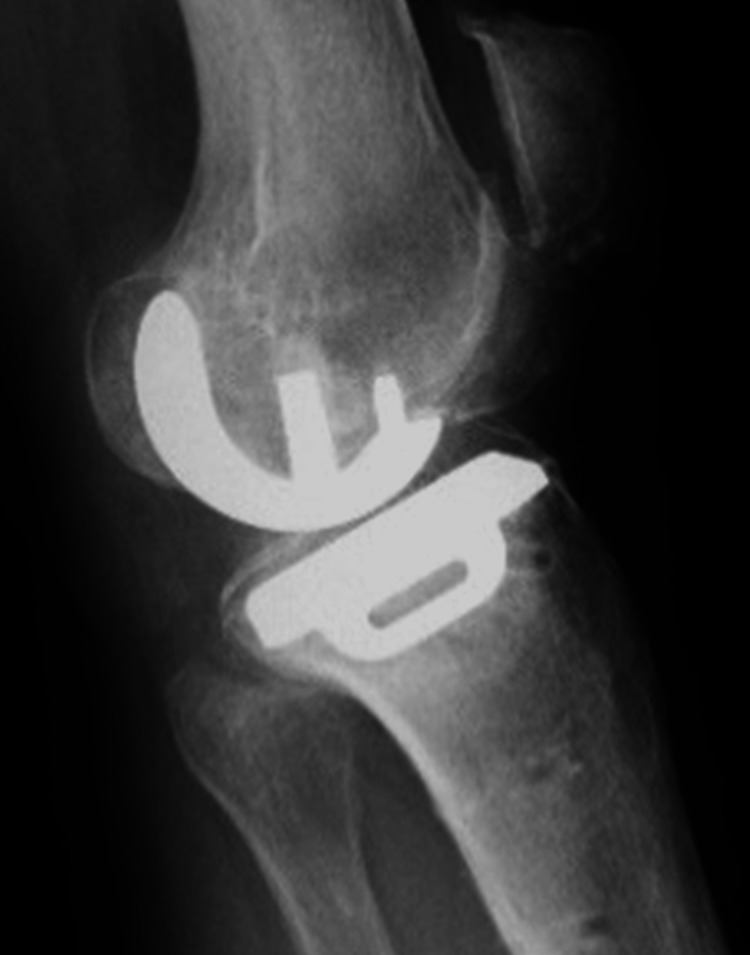
Postoperative radiography after plate removal (lateral view)

**Figure 17:**
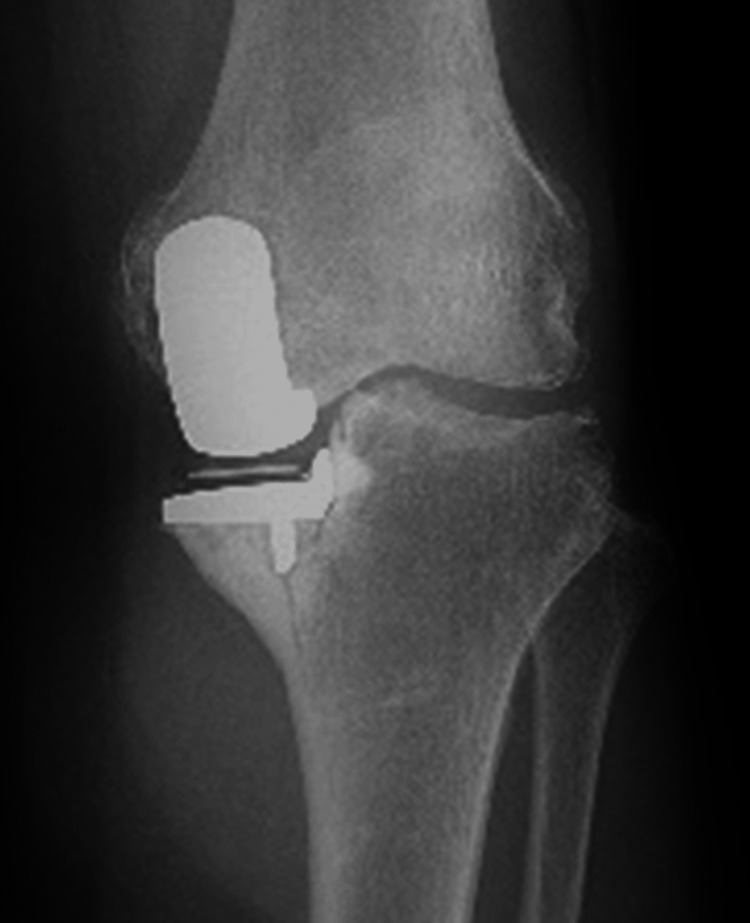
Radiography of refracture after plate removal (AP view)

**Figure 18:**
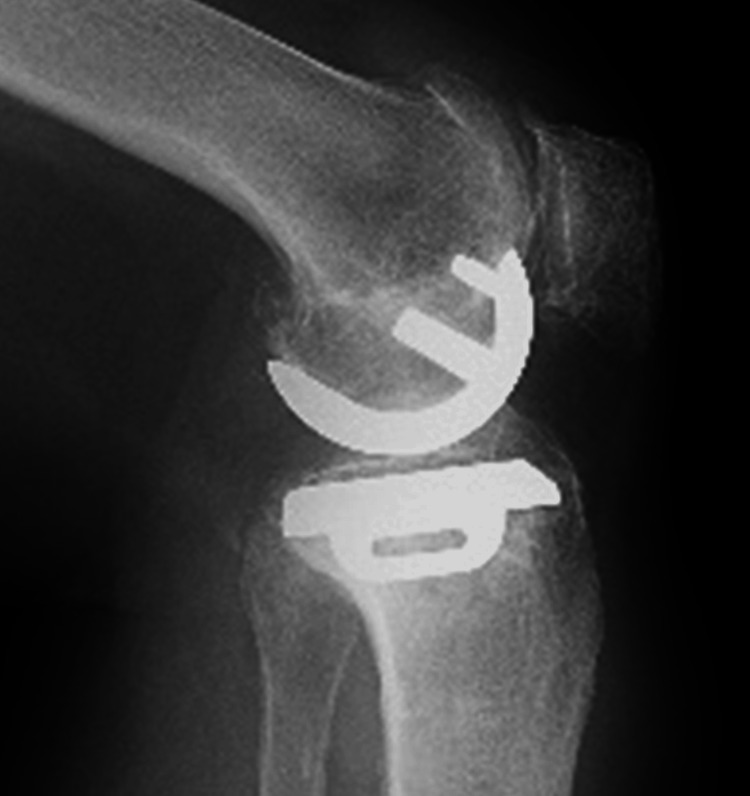
Radiography of refracture after plate removal (lateral view)

**Figure 19:**
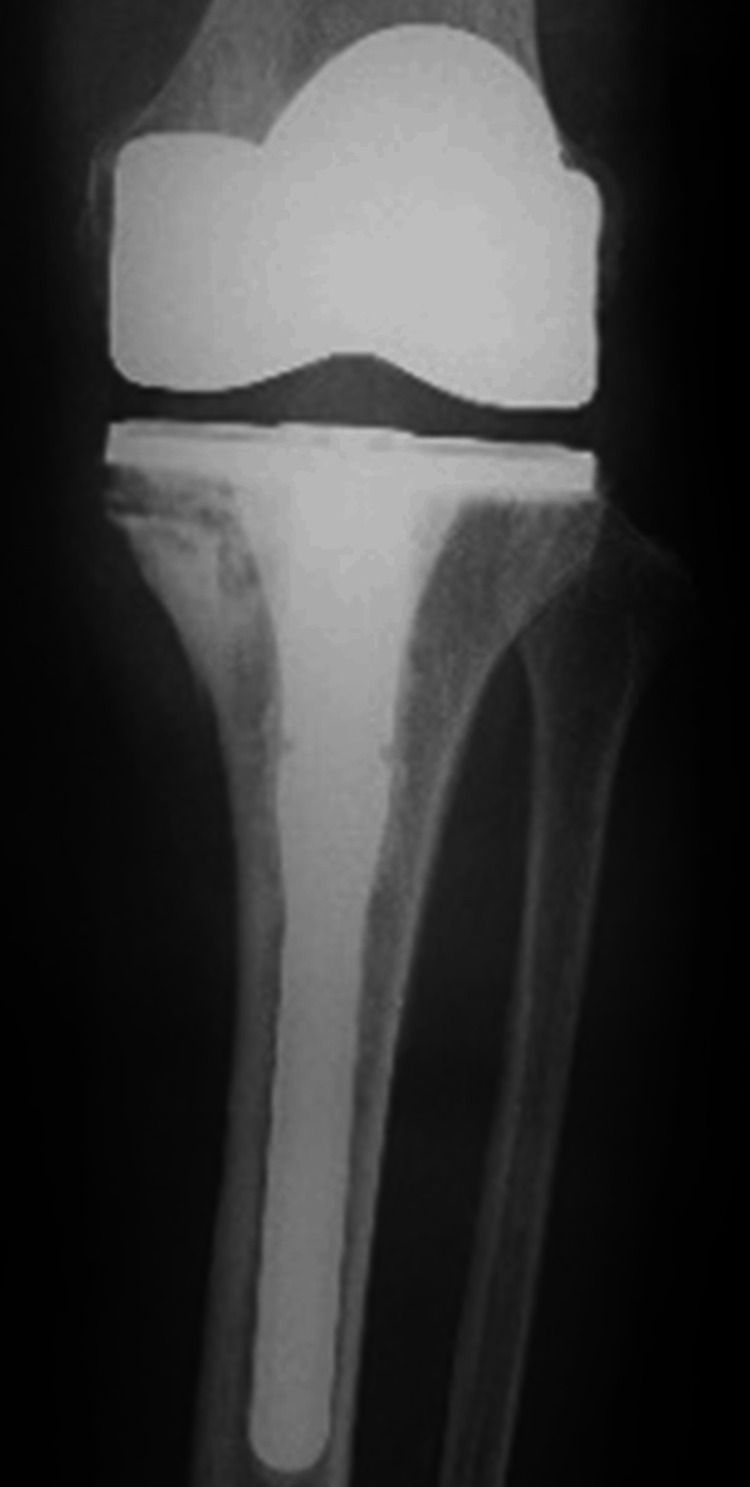
Radiography after conversion to TKA (AP view) TKA: total knee arthroplasty, AP: anteroposterior.

**Figure 20:**
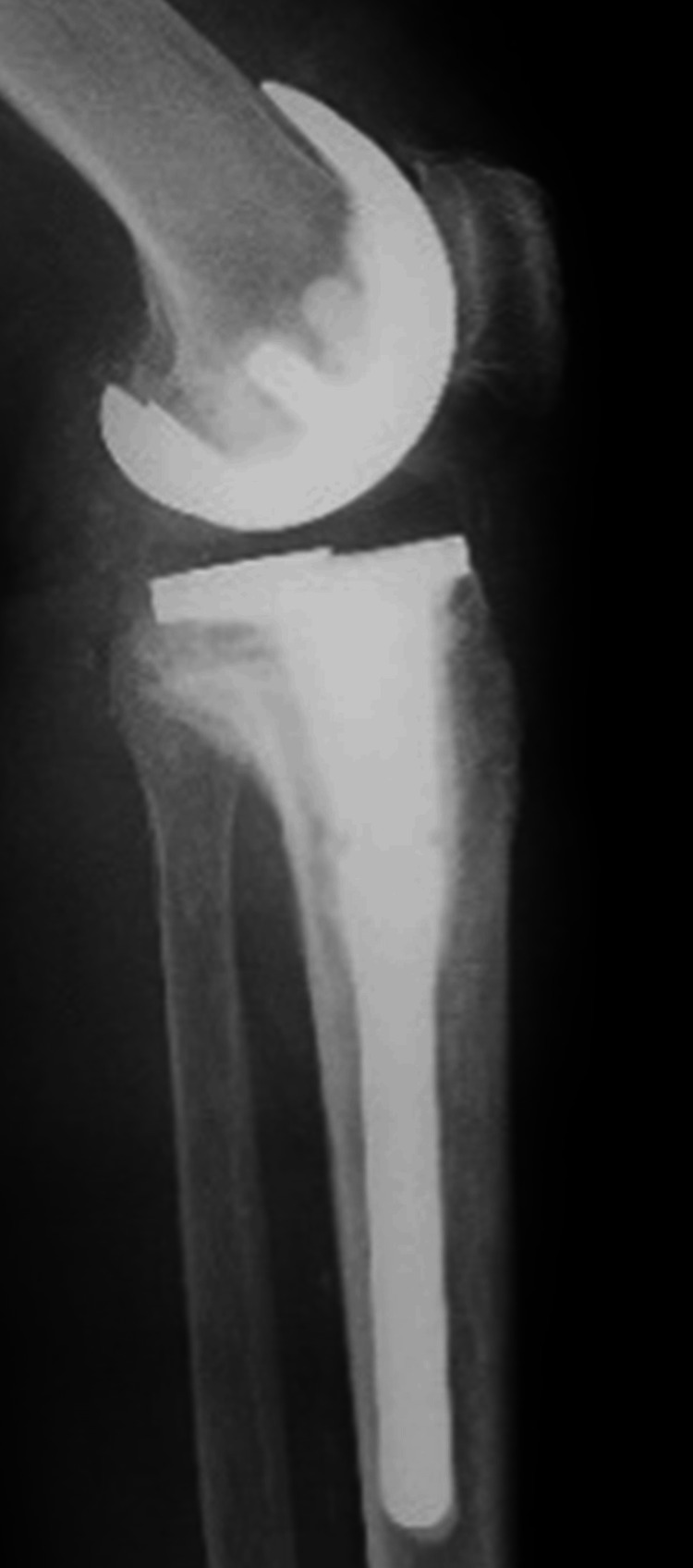
Radiography after conversion to TKA (lateral view) TKA: total knee arthroplasty.

One year after internal fixation, fracture union was confirmed on both radiographs and CT. Because the patient complained of discomfort and implant prominence on the medial side of the knee, the plate was removed at her request (Figures 15, 16). Radiographs and CT obtained after plate removal also showed maintained fracture union. However, one week later, she developed left knee pain, and radiographs demonstrated a refracture (Figures 17, 18). We therefore performed TKA (Persona MC, Zimmer Biomet) with a tibial stem without any fixation of the fragment (Figures 19, 20). One year postoperatively, the patient had 0°-130° range of motion in the left knee, and her Oxford Knee Score was 31. The fracture was united, and there were no signs of loosening.

## Discussion

We reported two cases of delayed fracture after mobile-bearing UKA. There have been numerous studies regarding periprosthetic fracture after UKA, but most fractures occurred intraoperatively or within approximately three months [1]. In one study, medial tibial collapse with medial tibial plateau fracture seven years after UKA was converted to TKA with a metal block and tibial stem, but the report lacked detail. This is therefore the first report to detail delayed periprosthetic fracture after UKA.

No evident cause of fracture was identified in either of our two cases. In previous reports, causes of acute fracture have included forced hammering, improper pinholes, and an extended sagittal saw [2, 3, 5, 7, 11, 13]. However, we believe none of these were causes in our cases because the fractures in this report occurred four and seven years after surgery, respectively. Possible causes include a decrease in bone strength compared with that at the time of surgery due to age-related progression of osteoporosis and stress shielding by the tibial implant. In addition to annual bone loss, stress shielding is another possible cause of delayed fracture, a well-known phenomenon after total hip arthroplasty. Reduced mechanical stress on the bone around the implant is a reported cause of stress shielding [7]. The rate of loss of bone mineral density progressively increased with age, i.e., -0.6%, -1.1%, and -2.1% per year for the 60-69, 70-79, and ≥80 age groups, respectively. As a consequence of this reduced loading, bone is lost and there is a decrease in bone density in the "shielded" region next to the implant. Stress shielding after knee arthroplasty has nonetheless not been widely reported. One finite element model analysis study showed that stress shielding can occur after TKA and is more evident when there is a thicker tibial tray [5]. Bone loss in a zonal pattern occurs in the first two years after TKA [14, 15]. Similarly, bone density around the tibial tray has been reported to decrease after UKA. Despite the possible bone loss around the tibial component, delayed fracture is rare, so other factors should be considered. The factors remain unclear, however, so careful observation is required.

In Case 2, internal fixation was performed, and fracture union appeared to have been achieved. However, refracture was observed immediately after plate removal. Although no obvious nonunion was identified on radiographs, incomplete fracture union may have remained. Unlike acute fractures, the underlying cause of delayed fractures is unclear and may persist even after internal fixation. Therefore, careful follow-up is needed after internal fixation for delayed peri-implant fractures after UKA. Therefore, treatment should be individualized according to fracture pattern, implant stability, bone quality, and patient factors.

After conversion to TKA, both patients have had good clinical courses. The use of a stem and the good quality of the lateral condyle seem to provide sufficient stability. Stemmed TKA has been shown to be a reliable solution because it increases stability by decreasing micromotion, provides shear resistance, and reduces tibial lift-off and the chance of aseptic loosening of the implant [16]. Interestingly, the fractures in both cases united uneventfully despite there being no internal fixation. This suggests that stability is sufficient with stemmed TKA [17]. We therefore also recommend stemmed TKA for delayed fractures.

## Conclusions

We reported two cases of delayed tibial peri-implant fractures after medial UKA. Although no definitive cause was identified, age-related deterioration in bone quality and stress shielding may have contributed to these fractures. Internal fixation alone may be insufficient in selected delayed cases, particularly when the fracture involves the tibial keel and is substantially displaced or when bone quality is poor. Stemmed TKA may be a useful treatment option because it can provide stable mechanical support and bypass the fracture site. Further studies are needed to determine the optimal treatment strategy for delayed peri-implant fractures after UKA.
